# Social Media Perceptions and Internet Verification Skills Associated With Human Papillomavirus Vaccine Decision-Making Among Parents of Children and Adolescents: Cross-sectional Survey

**DOI:** 10.2196/38297

**Published:** 2022-09-14

**Authors:** Erika L Thompson, Sharice M Preston, Jenny K R Francis, Serena A Rodriguez, Sandi L Pruitt, James-Michael Blackwell, Jasmin A Tiro

**Affiliations:** 1 Department of Biostatistics & Epidemiology School of Public Health University of North Texas Health Science Center Fort Worth, TX United States; 2 Center for Pediatric Population Health University of Texas Health Science Center at Houston School of Public Health Dallas, TX United States; 3 Department of Health Promotion & Behavioral Science University of Texas Health Science Center at Houston School of Public Health Houston, TX United States; 4 Department of Pediatrics University of Texas Southwestern Medical Center; Children’s Health Dallas, TX United States; 5 Department of Health Promotion & Behavioral Science University of Texas Health Science Center at Houston School of Public Health Dallas, TX United States; 6 Department of Population and Data Sciences University of Texas Southwestern Medical Center Dallas, TX United States; 7 Harold C Simmons Comprehensive Cancer Center University of Texas Southwestern Medical Center Dallas, TX United States

**Keywords:** HPV vaccination, human papillomavirus, social media, decision-making, vaccination, teens, adolescents, parent, USA, United States, misinformation, internet, survey, unvaccinated, child, online, health, literacy, decision, health care, decision, teen, vaccine

## Abstract

**Background:**

Human Papillomavirus (HPV) vaccination is recommended for children aged 11-12 years in the United States. One factor that may contribute to low national HPV vaccine uptake is parental exposure to misinformation on social media.

**Objective:**

This study aimed to examine the association between parents’ perceptions of the HPV vaccine information on social media and internet verification strategies used with the HPV vaccine decision-making stage for their child.

**Methods:**

Parents of children and adolescents aged 9-17 years were recruited for a cross-sectional survey in North Texas (n=1192) and classified into 3 groups: children and adolescents who (1) were vaccinated, (2) unvaccinated and did not want the vaccine, and (3) unvaccinated and wanted the vaccine. Multinomial logistic regression models were estimated to identify factors associated with the HPV vaccine decision-making stage with children and adolescents who were vaccinated as the referent group.

**Results:**

Of the 1192 respondents, 44.7% (n=533) had an HPV-vaccinated child, 38.8% (n=463) had an unvaccinated child and did not want the vaccine, and 16.4% (n=196) had an unvaccinated child and wanted the vaccine. Respondents were less likely to be “undecided/not wanting the vaccine” if they agreed that HPV information on social media is credible (adjusted odds ratio [aOR] 0.40, 95% CI 0.26-0.60; *P*=.001), disagreed that social media makes them question the HPV vaccine (aOR 0.22, 95% CI 0.15-0.33; *P*<.001), or had a higher internet verification score (aOR 0.74, 95% CI 0.62-0.88; *P*<.001).

**Conclusions:**

Interventions that promote web-based health literacy skills are needed so parents can protect their families from misinformation and make informed health care decisions.

## Introduction

Human papillomavirus (HPV) causes 34,800 anogenital and oropharyngeal cancer cases in the United States annually [1]. To prevent these cancer cases, the HPV vaccine is recommended for children aged 11-12 years. Additionally, unvaccinated persons can receive catch-up vaccination until the age of 26 years or participate in shared decision-making with a provider until the age of 45 years [2]. Despite this evidence-based recommendation, the rate of HPV vaccination is suboptimal. Healthy People 2030 aims to achieve a rate of 80% HPV vaccination coverage for adolescents [3], and although gains have been steady over the years, only 58.6% were up to date as of 2020 [4].

Parental exposure to health information on the internet and social media platforms may influence HPV vaccine awareness, decisions, and uptake. Most parents use the internet to search for information regarding their child’s health, especially to help prepare for questions when seeing a doctor [5]. In a study on Google searches related to preventable infectious diseases, looking for vaccine information generally was not prevalent; however, the HPV vaccine was the exception [6]. Thus, exposure to web-based content regarding HPV vaccination may be common for some parents prior to discussing with health care providers. Furthermore, a North Carolina study found that parents who learned of the HPV vaccine on the internet were more willing to get their daughters vaccinated [7]. Similarly, adult internet users were more likely to be aware of the HPV vaccine compared to noninternet users [8].

Despite being a source of factual information regarding HPV vaccination, social media and internet sources can increase exposure to *misinformation* (ie, false information aiming to deceive the reader [9]). From 2014-2017, Twitter bots (ie, Twitter accounts that are automated to post content and create impressions) were used to spread vaccine misinformation on social media platforms [10]. HPV vaccine content on social media is often user-generated [11] and includes positive (in favor of vaccines) and negative (against vaccines) messages [12-18], which can mean that parents, children, and adolescents are exposed to a variety of content on social media—some of which is not credible.

Health literacy and internet verification skills may improve information seeking and help counteract the spread of misinformation. Health literacy refers to how a person accesses, understands, appraises, and uses health information [19]. Internet verification skills may assist in identifying the veracity of information [19]. Given the expansive amounts of misinformation and negative information about vaccines on social media [20], especially on HPV vaccination [13,17], internet verification skills assessing content and source may help individuals better distinguish between credible and noncredible sources. Developing strategies to combat misinformation and increase confidence in the HPV vaccine via social media is a goal for HPV vaccine–related research [21]. In this study, we examined the association between parents’ perceptions of HPV vaccine information on social media and internet verification strategies used with the HPV vaccine decision-making stage for their child.

## Methods

### Sample and Data Collection

We used a purposive sample of parents of children and adolescents aged 9-17 years residing in 13 counties in North Texas. We contracted with 2 survey sampling and administration companies, 2M Research and Qualtrics, to field web-based surveys in English and Spanish. Both companies worked with third party vendors (eg, Marketing System Group and Poll Pay) to sample participants with children and adolescents aged 9-17 years residing in the 13-county catchment areas. Sample sizes for each county were based on county population densities. We used 2 different companies because they deployed different recruitment strategies to ensure a diverse sample. 2M Research mailed potential participants letters written in both English and Spanish introducing the study and directing the parent to the web-based survey URL. Qualtrics pushed the survey link via email to research panel participants. Data were collected in 2018.

The 80-item survey assessed factors hypothesized to influence HPV vaccine decision-making and vaccine hesitancy. Before beginning the survey, parents were oriented to the study and that continuing on to answer questions indicated consent. If parents reported having more than 1 child, the survey instructed them to complete the survey for the child whose age was closest to 11 years. The survey took approximately 15-20 minutes to complete. Only participants who completed the survey were included in the final analysis. Participants received a US $25 gift card.

### Ethics Approval

The University of Texas Southwestern Medical Center Institutional Review Board approved this study (STU 092017-076).

### Measures

The outcome variable was parental HPV vaccine decision-making for their child (see Multimedia Appendix 1 for specific items). This variable was operationalized as children and adolescents who were (1) already vaccinated, (2) unvaccinated and the parent was not aware, undecided, or did not want the HPV vaccine, and (3) unvaccinated and the parent wanted the HPV vaccine. This operationalization follows the World Health Organization’s definition of vaccine hesitancy incorporating behaviors and attitudes [22]. Our analysis retained the subgroup of parents who accepted HPV vaccination for their child, which enabled comparisons among the 3 groups.

Independent variables included those related to perceptions about information on social media, trust in providers, internet verification skills, and demographics. Respondents specified their level of agreement to 2 statements regarding HPV vaccine information on social media (“is credible” and “makes me question the HPV vaccine”; see Multimedia Appendix 1). Due to the data distribution, response categories were collapsed from a 5-point Likert scale into 3 categories: strongly agree/agree, neutral, and disagree/strongly disagree. The “completely trust the doctor or nurse’s judgement about my child’s medical care” item was categorized as trust (strongly agree and agree) and distrust (neutral, disagree, and strongly disagree). Internet verification behaviors was measured with 9 items [23,24] on a frequency Likert scale (see Multimedia Appendix 1 [23,24] for details). Items were summed (range 0-9) with higher scores indicating more performance of verification skills (Cronbach α=.92) [23,24]. Demographic variables included the sex and age of the parent and child, parent’s race/ethnicity, parent’s educational attainment, the number of children, and the type of residence (rural, urban, or suburban).

### Data Analysis

The distribution of participant characteristics was reported with descriptive statistics, stratified by child HPV vaccination status. All testing across child HPV vaccine status was reported with descriptive statistics, where the chi-square (categorical data) or Kruskal-Wallis (continuous data) test was used as appropriate. The Dwass-Steel-Critchlow-Fligner method was used for multiple comparisons testing. Univariate and multivariate multinomial logistic regressions were performed to identify factors associated with the 3-category HPV vaccine decision stage (children and adolescents who were vaccinated [referent], unvaccinated and did not want the HPV vaccine or was undecided, or unvaccinated and wanted the HPV vaccine). All data analysis was performed using SAS statistical software (version 9.4; SAS Institute).

## Results

### Sample Description

Overall, 1192 parents responded to the survey (Table 1). Among the 1192 parents, most were women (n=782, 65.6%), aged 35-44 years (n=518, 43.5%), who identified as white (n=716, 60.1%) and hold a college degree (n=747, 62.7%). Almost half (n=566, 47.5%) had a child aged 13-17 years and half (n=598, 50.2%) had 1 child. The participants resided across urban (n=471, 39.5%) and suburban (n=411, 34.5%) settings.

Most (n=1070, 89.8%) participants reported trusting their health care providers. With regard to social media, most were neutral about whether they perceived the HPV vaccination information on social media as credible (n=580, 48.7%) and were neutral about whether information on social media made them question the HPV vaccine (n=467, 39.2%). For HPV vaccination status, 533 (44.7%) parents had their child vaccinated for HPV, 463 (38.8%) had an unvaccinated child and did not want the vaccine, and 196 (16.4%) had an unvaccinated child and wanted the vaccine. The HPV vaccine decision stage was significantly associated with the parent’s gender (*P*<.001), the parent’s age (*P*=.02), the child’s age (*P*<.001), the number of children (*P*=.007), trust in health care providers (*P*<.001), the credibility of HPV vaccine information on social media (*P*<.001), information on social media making them question HPV vaccination (*P*<.001), and internet verification behaviors (*P*<.001).

**Table 1:** Descriptive characteristics of parents of children and adolescents from the Dallas-Fort Worth area by human papillomavirus (HPV) vaccine decision-making status (N=1192).

Characteristic			Vaccinated^a^ (n=533)		Unvaccinated did not want the vaccine^a^ (n=463)		Unvaccinated and wanted the vaccine^a^ (n=196)		Total (N=1192)		*P* value	
**Parent’s gender, n (%)**												<.001
	Female	325 (61)		298 (64.4)		158 (80.6)		782 (65.6)				
	Male	208 (39)		164 (35.4)		37 (18.9)		409 (34.3)				
**Parent’s age (years), n (%)**												.02
	18-24	15 (2.8)		14 (3)		7 (3.6)		36 (3)				
	25-34	52 (9.8)		72 (15.6)		35 (17.9)		159 (13.3)				
	35-44	228 (42.8)		208 (44.9)		82 (41.8)		518 (43.5)				
	45-54	184 (34.5)		142 (30.7)		53 (27)		380 (31.9)				
	55-64	48 (9)		22 (4.8)		17 (8.7)		87 (7.3)				
	≥65	6 (1.1)		3 (0.6)		2 (1)		11 (0.9)				
**Child’s age (years), n (%)**												<.001
	<11	71 (13.3)		154 (33.3)		84 (42.9)		310 (26)				
	11-12	133 (25)		127 (27.4)		57 (29.1)		317 (26.6)				
	13-17	329 (61.7)		182 (39.3)		55 (28.1)		566 (47.5)				
**Parent’s race, n (%)**												.14
	White	325 (61)		264 (57)		127 (64.8)		716 (60.1)				
	Non-White	208 (39)		198 (42.8)		68 (34.7)		475 (39.8)				
**Parent’s education, n (%)**												.11
	Did not attend college	84 (15.8)		101 (21.8)		35 (17.9)		220 (18.5)				
	Some college	97 (18.2)		91 (19.7)		36 (18.4)		224 (18.8)				
	College graduate	352 (66)		270 (58.3)		124 (63.3)		747 (62.7)				
**County type, n (%)**												.39
	Urban	227 (42.6)		173 (37.4)		71 (36.2)		471 (39.5)				
	Suburban	177 (33.2)		162 (35)		72 (36.7)		411 (34.5)				
	Other	129 (24.2)		128 (27.6)		53 (27)		311 (26.1)				
**Number of children, n (%)**												.007
	1	279 (52.4)		228 (49.2)		91 (46.4)		598 (50.2)				
	2	194 (36.4)		167 (36.1)		82 (41.8)		443 (37.2)				
	3	32 (6)		55 (11.9)		18 (9.2)		106 (8.9)				
	4	8 (1.5)		5 (1.1)		4 (2)		17 (1.4)				
	5	20 (3.8)		8 (1.7)		1 (0.5)		29 (2.4)				
**Trust in providers, n (%)**												<.001
	Trust providers	516 (96.8)		370 (79.9)		183 (93.4)		1070 (89.8)				
	Distrust providers	17 (3.2)		93 (20.1)		13 (6.6)		123 (10.3)				
**HPV information on social media is credible, n (%)**												<.001
	Agree/strongly agree	226 (42.4)		96 (20.7)		50 (25.5)		372 (31.2)				
	Neutral	217 (40.7)		259 (55.9)		104 (53.1)		580 (48.7)				
	Disagree/strongly disagree	90 (16.9)		107 (23.1)		42 (21.4)		240 (20.1)				
**Information on social media makes me question the HPV vaccine, n (%)**												<.001
	Agree/strongly agree	162 (30.4)		150 (32.4)		29 (14.8)		341 (28.6)				
	Neutral	155 (29.1)		236 (51)		76 (38.8)		467 (39.2)				
	Disagree/strongly disagree	216 (40.5)		75 (16.2)		91 (46.4)		383 (32.1)				
Internet verification scale^b^, median (IQR)			3.9 (3.3-4.4)		3.6 (3.0-4.1)		3.8 (3.1-4.2)		3.8 (3.1-4.2)		<.001	

^a^Outcome groups: vaccinated for HPV; unvaccinated and did not want or undecided about HPV vaccination; and unvaccinated and wanted HPV vaccination.

^b^Scale: range 0-9; higher value=more internet verification skills used.

### Patterns of Association in the Multivariable Multinomial Model

Parents who were undecided or did not want their child to be vaccinated were compared to those with a vaccinated child. In the multivariable model (Table 2), the following characteristics were significantly associated with *increased* odds of being *undecided or not wanting the HPV vaccine* as compared to parents with a vaccinated child: having a child aged <11 years (vs aged 11-12 years; adjusted odds ratio [aOR] 2.38, 95% CI 1.56-3.63; *P*<.001); distrusting providers (vs those who trusted providers; aOR 6.37, 95% CI 3.58-11.32; *P*<.001); and disagreeing that HPV information on social media is credible (vs neutral; aOR 1.90, 95% CI 1.25-2.87; *P*=.002). Characteristics significantly associated with *decreased* odds of being *undecided/not wanting the vaccine* compared to parents with a vaccinated child included having a child aged 13-17 years (vs aged 11-12 years; aOR 0.53, 95% CI 0.37-0.76; *P*=.001); being a college graduate (vs not attending college; aOR 0.65, 95% CI 0.43-0.98; *P*=.04); agreeing that HPV information on social media is credible (vs neutral; aOR 0.40, 95% CI 0.26-0.60; *P*=<.001); disagreeing that social media made the parent question the HPV vaccine (vs neutral; aOR 0.22, 95% CI 0.15-0.33; *P*=<.001); and having a higher mean internet verification score (aOR 0.74, 95% CI 0.62-0.88; *P*=.001). Table 3 illustrates the consistent pattern of how the use of each verification skill is associated with a higher prevalence of having a vaccinated child than parents of an unvaccinated child who did not want the vaccine or were undecided.

Parents who wanted their child vaccinated were compared to those who have already vaccinated their child. In the multivariable model, the following characteristic was significantly associated with *increased odds* of *wanting the HPV vaccine* as compared to parents with a vaccinated child: having a child aged <11 years (vs aged 11-12 years; aOR 3.07, 95% CI 1.89-5.00; *P*<.001). Characteristics significantly associated with *decreased* odds of *wanting the vaccine* compared to parents with a child already vaccinated included being a male parent (vs female parent; aOR 0.42, 95% CI 0.27-0.64; *P*<.001); having a child aged 13-17 years (vs aged 11-12 years; aOR 0.34, 95% CI 0.21-0.54; *P*<.001); and agreeing that social media made parent question the HPV vaccine (vs neutral; aOR 0.41, 95% CI 0.23-0.74; *P*=.003). The mean internet verification scale was not significantly associated with parents wanting the HPV vaccine compared to the vaccinated group (*P*=.96).

**Table 2:** Multinomial logistic regression modeling of the human papillomavirus (HPV) vaccine decision-making stage among parents of children and adolescents in the Dallas-Fort Worth area (N=1192).

Characteristic			Unvaccinated and did not want the vaccine^a^, aOR^b^ (95% CI)		*P* value		Unvaccinated and wanted the vaccine^a^, aOR (95% CI)		*P* value
**Parent’s gender**									
	Female	Reference				Reference			
	Male	1.19 (0.87-1.62)		.28		0.42 (0.27-0.64)		<.001	
**Parent’s age (years)**									
	18-24	Reference				Reference			
	25-34	1.31 (0.51-3.37)		.57		1.06 (0.33-3.34)		.93	
	35-44	1.19 (0.49-2.90)		.70		0.76 (0.25-2.28)		.62	
	45-54	1.15 (0.46-2.87)		.77		0.74 (0.24-2.31)		.61	
	55-64	0.61 (0.22-1.74)		.36		1.12 (0.33-3.85)		.86	
	≥65	1.19 (0.20-7.18)		.85		1.51 (0.20-11.33)		.69	
**Child’s age (years)**									
	<11	2.38 (1.56-3.63)		<.001		3.07 (1.89-5.00)		<.001	
	11-12	Reference				Reference			
	13-17	0.53 (0.37-0.76)		<.001		0.34 (0.21-0.54)		<.001	
**Parent’s education**									
	Did not attend college	Reference				Reference			
	Some college	0.82 (0.51-1.32)		.42		0.78 (0.42-1.43)		.42	
	College graduate	0.65 (0.43-0.98)		.04		0.92 (0.55-1.55)		.76	
**Number of children**									
	1	Reference				Reference			
	2	1.03 (0.75-1.42)		.86		1.17 (0.79-1.74)		.43	
	3	1.42 (0.82-2.46)		.21		1.11 (0.56-2.22)		.77	
	4	0.88 (0.22-3.47)		.85		1.49 (0.39-5.74)		.57	
	5	0.51 (0.18-1.39)		.19		0.18 (0.02-1.49)		.11	
**Trust in providers**									
	Trust providers	Reference				Reference			
	Distrust providers	6.37 (3.58-11.32)		<.001		1.84 (0.83-4.07)		.13	
**County of residents**									
	Urban	Reference				Reference			
	Suburban	1.34 (0.95-1.89)		.10		1.17 (0.76-1.80)		.47	
	Other	1.30 (0.90-1.89)		.17		1.21 (0.76-1.92)		.42	
**HPV information on social media is credible**									
	Agree/strongly agree	0.40 (0.26-0.60)		<.001		0.64 (0.40-1.03)		.07	
	Neutral	Reference				Reference			
	Disagree/strongly disagree	1.90 (1.25-2.87)		.002		1.08 (0.65-1.79)		.77	
**Information on social media makes me question the HPV vaccine**									
	Agree/strongly agree	0.95 (0.64-1.41)		.80		0.41 (0.23-0.74)		.003	
	Neutral	Reference				Reference			
	Disagree/strongly disagree	0.22 (0.15-0.33)		<.001		0.98 (0.63-1.51)		.92	
Internet verification scale			0.74 (0.62-0.88)		.001		0.99 (0.80-1.24)		.96

^a^Reference group for outcomes: having a child who was vaccinated.

^b^aOR: adjusted odds ratio.

**Table 3:** Proportion of participants who report the use of internet verification skills every time/almost all the time by human papillomavirus (HPV) vaccination status among parents of children and adolescents in the Dallas-Fort Worth area (N=1192).

Internet verification skill	Vaccinated (n=533), n (%)	Unvaccinated and wanted the vaccine (n=196), n (%)	Unvaccinated and did not want the vaccine (n=463), n (%)	*P* value
Check if the website information is up to date	388 (72.8)	134 (68.4)	288 (62.2)	.005
Check if the website information is complete with all the need-to-know info	378 (70.9)	128 (65.3)	278 (60)	.007
Think about whether the writer is giving facts or opinion	402 (75.4)	140 (71.4)	298 (64.4)	<.001
Check other places to see if the information is true	387 (72.6)	140 (71.4)	310 (67)	.15
Think about why the author posted the information	297 (55.7)	95 (48.5)	208 (44.9)	.003
Check to see who wrote the website	326 (61.2)	104 (53.1)	236 (51)	.01
Look for recommendations from someone they know	289 (54.2)	83 (42.4)	209 (45.1)	.01
Check to see if the website or author gives contact information	268 (50.3)	92 (46.9)	151 (32.6)	<.001
Check to see if the author lists their expertise on the topic	336 (63)	113 (57.7)	246 (53.1)	.004

## Discussion

Prior to entering a physician’s office, parents may be exposed to information on HPV vaccination via the internet and social media. Although some information may be useful for informed decision-making on HPV vaccination, misinformation also exists [17]. This study explored how internet verification skills and perceptions of HPV vaccine information on social media relate to HPV vaccination and decision-making among parents of children and adolescents. Overall, we found that parents’ trust in providers, perceptions of HPV vaccine information credibility on social media, reporting that social media information makes one question HPV, and internet verification skills were related to *not* wanting HPV vaccination for their child. The parent’s gender, younger age of the child, and prompts for questioning HPV vaccination based on social media information were related to wanting the vaccine.

Parents of vaccinated children and adolescents reported performing more internet verification behaviors compared to parents in the unvaccinated and unwanted group. These behaviors included checking that the website is up to date and has a credible author and cross-checking with other sources. Our finding may explain why parents with a vaccinated child do not question information they see on social media, because they have the internet verification skills to filter through misinformation. Previous research has found that parents desire guidance on how to search and assess the reliability of information found on the internet [5]. Empowering parents with health literacy skills to filter health information on the internet, particularly related to vaccines, may be an important strategy to promote positive attitudes and intentions toward vaccination and, ultimately, HPV vaccine uptake.

Overall, many people find it difficult to distinguish credible and noncredible information sources [25]; however, this finding may be changing with exposure to more information on the vaccine development process with COVID-19 vaccines. The field needs to examine how COVID-19 vaccine–specific attitudes influence the attitudes and uptake of other vaccines. For example, parents with fewer internet verification skills may question the HPV vaccine more given the COVID-19 media coverage, which could result in additional questioning when discussing the HPV vaccine with providers [26]. The ubiquitous nature of social media results in high exposure to potential misinformation, which may increase parental hesitancy and potentially frustrate health care providers due to the challenges associated with managing patient concerns from social media sources. To encourage vaccination, providers must attend to parents’ concerns in a nonconfrontational and nonjudgmental manner with parents who question vaccines [26].

Provider recommendation and discussion are imperative to HPV vaccine initiation and completion among adolescents [27,28]. Although provider-patient communication is a component of most medical education curricula, some providers express low confidence in their ability to influence parents regarding vaccination [29]. In a recent study, about a third of providers reported that over 10% of parents of adolescents in their practice expressed HPV vaccine hesitancy, whereas over 50% of pediatricians in the same study did not feel confident responding to parents’ misinformation obtained from the internet/social media or the news [30]. The quality of provider recommendations has consistently predicted HPV vaccine initiation and completion, and multiple interventions to educate providers on reliable techniques (patient reminders, presumptive recommendation, and reference to HPV vaccination as cancer prevention) are available to support providers and reinforce vaccine communication skills [31,32].

Perceptions of credibility of social media HPV vaccination information is relevant for parents’ HPV vaccine decisions. Specifically, parents who did not believe that the information they saw on social media is credible were more likely to not want the vaccine. This finding may be attributed to the types of information parents are exposed to on social media. Although information on HPV vaccination on the internet is both positive and negative [17], social media algorithms and social networks may bias the types of information parents are exposed to so that it aligns with their beliefs. As such, additional research is needed to explore how an individual’s beliefs, health literacy skills, and information-seeking behaviors intersect with community and group norms driven by social media platforms. Moreover, researchers should test novel interventions that adapt messaging in real time based on evolving social media content; recent advances in artificial intelligence and machine learning are potential avenues moving forward. However, previous research has found that tools, such as web-based smart assistants, do not always provide credible HPV vaccine information [33]. There is also evidence that combatting misinformation in a “myth-versus-fact” format tends to backfire and reinforce the preexisting belief in the myth [34,35].

Similarly, parents who did not want the HPV vaccine were less likely to question the vaccine based on exposure to information on social media than the vaccinated group. In contrast, parents who wanted their child vaccinated were less likely to think the information on social media makes them question the HPV vaccine than parents with a vaccinated child. Thus, persons who do not intend to vaccinate their child for HPV may already be exposed to information that confirms their beliefs on vaccination, whereas persons who intend to vaccinate their children may not have enough information to transition to the vaccine decision-making stage. Social media users on Facebook and Twitter are likely to be exposed to like-minded posts via the echo chamber effect [36]. For example, parents with a vaccinated child could have been exposed to more pro-vaccine messages, which could heighten perceptions of credibility and lead to vaccination behaviors. Additional longitudinal studies are needed to examine the temporality of the types of information exposure on the internet/social media and future vaccine behavior. Moreover, as social media is used to share information, developing novel strategies to combat misinformation on various platforms is urgently needed. Promoting evidence-based information on vaccination on the internet and social media via trusted messengers, such as providers, may be an effective approach compared to the removal and censorship of anti-vaccine content alone [37]. Given that not all persons engage in internet verification skills when consuming health information, providers and other trusted messengers, such as other parents [38], could be an accurate dissemination channel on social media and the internet. This process would require the development of social media strategies to reach intended audiences and relying on algorithms so that the content is more prominent in search results and social media feeds. However, a recent study found that anti-vaccine social media posts are associated with increases in mothers’ general vaccine hesitancy and decreases in their children’s HPV vaccination rates, whereas pro-vaccine content were not associated with hesitancy nor vaccination rates [39]. As a whole, the literature on social media and HPV vaccination is in its infancy, and a recent systematic review by Ortiz et al [40] recommends more rigorous and systematic research.

Finally, another key finding was that parents who did not want their child vaccinated for HPV were more likely to distrust providers than parents who vaccinated their child. Taken in context with other study findings, the parents who do not want their child vaccinated may be going to social media to corroborate their beliefs or are exposed to misinformation on the internet contributing to their beliefs. Studies are needed to experimentally test how exposure to misinformation and correct information on social media influences decisions for vaccination, and how and who is best to intervene in this evolving setting. Ultimately, a segmented approach to vaccine information dissemination is needed to reach different parental groups on the hesitancy spectrum.

These findings should be recognized in the context of study limitations. First, this study was cross-sectional, and we could not assess the temporality between exposure to information on social media, internet verification skills, and the vaccine decision-making stage. As such, respondents may have adopted attitudes that align with their current behavior to reduce cognitive dissonance. Second, these data were derived from a sample in North Texas and may not be generalizable to other US regions. Additionally, HPV vaccination status was self-reported, and misclassification bias for the outcome variable may be present. Finally, these data were collected prior to the COVID-19 pandemic, and perceptions regarding social media and credibility may have shifted. Internet verification skills and strategies, however, could similarly impact COVID-19 vaccine decision-making. These findings could be relevant to apply toward vaccine hesitancy studies about COVID-19.

Although many strategies to promote HPV vaccination have focused on the provider recommendation during a visit, extensive exposure to social media before a visit may inform parents’ beliefs and attitudes toward HPV vaccination and, ultimately, their decision to vaccinate their child. Thus, interventions that promote web-based health literacy skills are needed so that parents can make informed health care decisions with their providers. Social media will remain an ongoing obstacle to evidence-based health information, and public health responses must adapt to this challenge accordingly.

## Appendix

Multimedia Appendix 1 Survey items used in the analysis.

## Abbreviations

aOR: adjusted odds ratio
HPV: human papillomavirus
