# Supplementary material for: Social Media Perceptions and Internet Verification Skills Associated With Human Papillomavirus Vaccine Decision-Making Among Parents of Children and Adolescents: Cross-sectional Survey
Source: JMIR Pediatr Parent. 2022 Sep 14;5(3):e38297. doi: 10.2196/38297 (PMC9520398; doi:10.2196/38297)
Supplement: Multimedia Appendix 1 [file pediatrics_v5i3e38297_app1.docx]

**Appendix 1. Survey items used in the analysis**

This is a subset of items from an 80-item survey examining factors associated with HPV vaccination, decision stage, and vaccine hesitancy among parents residing in North Texas.

***HPV vaccine decision-stage***

“This variable was operationalized as (1) adolescent already vaccinated (2) unvaccinated and parent is not aware, undecided or does not want the HPV vaccine, and (3) unvaccinated and parent wants it.” The following items were combined:

1. Has your [xx] year old [son/daughter] ever received HPV shots?

- Yes
- No
- I don't know

1. How many HPV shots did [he/she] receive? (*Not used for this analysis)*

## of shots

- I don't know

1. Which statement best describes your thoughts about getting the HPV vaccine for your [XX] year old [son/daughter]?

- I have never thought about getting the HPV vaccine for him/her.
- I am undecided about getting the HPV vaccine for him/her.
- I do not want to get the HPV vaccine for him/her
- I do want to get the HPV vaccine for him/her

***Perceptions about information on social media***

How much do you agree or disagree with these statements:

1. HPV vaccination information I see on social media is credible.
2. Information I see on social media makes me question the HPV vaccine.

Response options:

- Strongly agree
- Agree
- Neither
- Disagree
- Strongly disagree

***Trust in provider***

How much do you agree/disagree with this statement:

1. I completely trust the doctor or nurse's judgment about my child's medical care.

***Internet verification Skills^a^***

When you go to a website, how often do you do the following?

1. Check to see if the information is up-to-date.
2. Check to see if the information is complete and includes all you would need to know.
3. Think about whether the writer is giving facts or opinions.
4. Check other places to see if the information is true.
5. Think about why the author posted the information online.
6. Check to see who wrote the website.
7. Look for a recommendation from someone I know.
8. Check to see if the site or author gives contact information.
9. Check to see if the author lists their expertise on the topic.

Response options:

- Never
- Almost never
- Sometimes
- Most of the time
- All of the time

*^a^Question Sources:*

Flanagin AJ, Metzger MJ. Perceptions of Internet Information Credibility. Journalism & Mass Communication Quarterly. 2000;77(3):515-40. doi: 10.1177/107769900007700304.

Metzger MJ. Making sense of credibility on the Web: Models for evaluating online information and recommendations for future research. Journal of the American Society for Information Science and Technology. 2007;58(13):2078-91. doi: 10.1002/asi.20672.
